# Relative Age Effect in Olympic Karate: Evidence from Tokyo 2020

**DOI:** 10.3390/jfmk10040456

**Published:** 2025-11-21

**Authors:** Sofia Serafini, Simone Ciaccioni, Gabriele Mascherini, Pascal Izzicupo

**Affiliations:** 1 Department of Medicine and Aging Sciences, University “G. D’Annunzio” of Chieti-Pescara, 66100 Chieti, Italy; sofia.serafini@phd.unich.it (S.S.); pascal.izzicupo@unich.it (P.I.); 2 Department of Neurosciences, Biomedicine and Movement Sciences, University of Verona, 37124 Verona, Italy; 3 Department of Education and Sport Sciences, Pegaso Telematic University, 80143 Naples, Italy; 4 Department of Movement, Human and Health Sciences, University of Rome “Foro Italico”, 00135 Rome, Italy; 5 Department of Experimental and Clinical Medicine, University of Florence, 50134 Florence, Italy

**Keywords:** RAE, combat sports, martial arts, kumite, kata, Tokyo 2020, sex differences, talent identification, maturity, élite athletes

## Abstract

**Background:** The Relative Age Effect (RAE) refers to the advantage conferred to athletes born earlier within a selection year. In karate, particularly at the highest level, evidence is lacking. This study aimed to examine the presence of RAE among male and female karate athletes competing at the Tokyo 2020 Olympic Games, in two competitive disciplines: kata and kumite. **Methods:** Data from 81 athletes (42 males, 39 females) were retrieved from open-access databases. Birthdates were grouped into quartiles (Q1–Q4) and semesters (S1–S2). Chi-squared tests and odds ratios (ORs) were used to assess deviations from a uniform distribution, while binary logistic regression examined the association between semester of birth and medal attainment. **Results:** The overall distribution of birth quartiles significantly deviated from a uniform distribution (χ^2^(3) = 9.81, *p* = 0.020), indicating a higher proportion of athletes born in Q1 (38%) compared with Q4 (19%; OR = 2.07). RAE was particularly evident in kumite (χ^2^(3) = 17.87, *p* < 0.001; OR = 3.50 for Q1 vs. Q4) and among female athletes (χ^2^(3) = 9.92, *p* = 0.019), whereas no significant effect was found in kata or among males. Logistic regression revealed no significant association between semester of birth and medal success (OR = 0.49, 95% CI [0.20–1.21], *p* = 0.125). **Conclusions:** This study provides the first evidence of RAE in Olympic karate, especially among females and in kumite. However, relative age did not predict performance outcomes, suggesting that once athletes reach the Olympic level, technical and tactical factors outweigh birthdate advantages.

## 1. Introduction

The Relative Age Effect (RAE) describes the competitive advantage that athletes born in the early months of the selection year may gain over those born later in the same year [1]. This phenomenon is derived from the cut-off criterion, which is generally set for the calendar year. The primary purpose of these cut-offs is to provide for fair competition and equal opportunities, although they can result in an age gap of up to 12 months between competitors [2,3]. This age difference may lead to advantages during the selection process, mediated by greater height, body mass, muscular development, and more advanced physical and motor capacities, which shape the way athletes are preferentially identified and retained [4,5,6]. Relatively older athletes, therefore, tend to benefit from better selection opportunities, greater competitive experience, and faster technical and tactical development [4,7]. As a result, RAE has been documented across numerous sports, both team-based and individual [8,9].

Combat sports present peculiar characteristics compared to other disciplines, since performance depends not only on technical–tactical and psychological skills but also on athletes’ physical and conditioning capacities [10,11,12,13]. In direct confrontation, a stronger and more robust athlete may hold an immediate advantage, particularly in phases of contact and opposition. In this context, the literature on RAE has produced heterogeneous results. In judo, a retrospective analysis of Olympic athletes from 1964 to 2012 revealed evidence of RAE, especially among males, with a particular incidence in heavyweight categories and among medalists [14]. In Olympic wrestling, RAE was observed across all styles, particularly among men, suggesting that weight categories are insufficient to neutralize the effect [15]. In taekwondo, by contrast, an analysis of athletes competing in the Atlanta, Sydney, and Beijing Games found no significant differences in quartile distribution, indicating no clear evidence of RAE [16]. More recently, studies in international boxing reported a pronounced RAE in males, particularly in youth categories. In females, distinct trends were observed, with relatively older athletes being more prevalent among young participants and a higher proportion of athletes born in the later quartiles among medalists [17]. This variability likely reflects the spectrum of combat sports, ranging from strength-based grappling disciplines to striking ones relying on speed, reactivity, and timing, such as taekwondo and karate, all of which can nonetheless be affected by relative age advantages.

Karate is a traditional Japanese martial art that has evolved into a modern competitive sport, combining striking techniques with elements of timing, precision, and strategy [18]. In its Olympic format, introduced for the first time at Tokyo 2020, karate comprised two distinct events. Kata consists of the performance of predetermined sequences of offensive and defensive techniques executed at maximal intensity and assessed by judges according to technical execution and athletic criteria [19]. Kumite, in contrast, is a semi-contact combat discipline in which two athletes face each other under a points-based system, with scoring awarded for controlled and effective techniques such as ippon, waza-ari, and yuko (3, 2, and 1 point(s), respectively) [18,20]. While kata emphasizes technical precision, balance, and expression, kumite places greater demands on speed, reactivity, and timing capacities closely related to neuromuscular development and perceptual–motor coordination [21].

Despite the growing body of research on RAE in combat sports, to the best of our knowledge, only a few studies have investigated karate at the national level [22,23]. This represents a relevant gap, as karate was included for the first time in the Olympic program at Tokyo 2020, offering a unique opportunity to explore the dynamics of age-related selection and representation in a discipline characterized by two distinct competitive formats (kata and kumite). The aim of the present study was to investigate the presence of RAE in male and female Olympic karate athletes by analyzing the birth quartile distribution among participants in the Tokyo 2020 Games. It was hypothesized that the distribution would not be uniform but instead show an over-representation of athletes born in the first quartile and an under-representation of those born in the last quartile.

## 2. Materials and Methods

### 2.1. Participants and Data Collection

A total of 81 athletes (males: 42, females: 39) were included in the overall analysis. The mean age of participants was 28.22 ± 4.36 years for males and 28.01 ± 4.43 years for females. Athlete data, including name, date of birth, sex, nationality, discipline (i.e., kata, kumite), weight category, and competition result, were retrieved from online, open-access, publicly available databases [24,25]. This procedure has been validated in previous studies that investigated the RAE using publicly available datasets [16,17,26,27]. It is noteworthy that the use of public databases exempts the present study from review by a local ethics committee. Additionally, the present study adhered to the STROBE guidelines for observational studies (Table S1) [28].

### 2.2. Procedure

Websites were accessed to collect athletes’ data. Then, dates of birth were grouped into four quartiles: Q1 (January–March), Q2 (April–June), Q3 (July–September), and Q4 (October–December). Data were entered manually into Microsoft Excel (Version 16.101.1, Microsoft Corporation, Redmond, WA, USA, 2025) for organization and verification.

### 2.3. Statistical Analysis

To evaluate deviations from the expected distribution of births across quartiles, chi-squared (χ^2^) goodness-of-fit tests were performed. As in other RAE studies involving international samples, the expected values were based on a uniform distribution (25% per quartile) [17,26]. Odds ratios (ORs) were calculated to compare the relative frequency of births in Q1, Q2, and Q3 against Q4, which was used as the reference category. Analyses were conducted for the total sample and stratified by sex and discipline. For the overall sample and male and female subgroup comparisons, the minimum expected count of five observations per subgroup was met, allowing for stable statistical estimates. In contrast, for kata and male kumite athletes, analyses must be considered approximate due to one expected cell frequency < 5. For male and female kata athletes and female kumite athletes, subgroup analyses were not performed due to more than one expected cell frequency < 5. An additional examination was conducted by aggregating quartiles into semesters (S1 = Q1–Q2; S2 = Q3–Q4) when one or more expected cell frequency was <5 (overall kata, male kumite, female kumite, male kata, and female kata). This procedure, aimed at ensuring adequate statistical power and model stability, follows prior RAE research where quartiles were aggregated into semesters to manage small sample sizes and maintain robust estimations [29]. Binary logistic regression analyses were performed to assess the likelihood of winning a medal versus being eliminated for the overall sample, using S2 as the reference category. This approach was adopted because aggregation into semesters ensured model stability. ORs and 95% confidence intervals (CIs) were reported to estimate differences in medal attainment between semesters. The level of statistical significance was set at *p* < 0.05. All data processing and statistical analyses were performed using R statistical software (Version 4.3.1; R Core Team, Vienna, Austria, 2023).

## 3. Results

### 3.1. Overall Effect

The distribution of birth quartiles was as follows: Q1 = 38.3%, Q2 = 16.0%, Q3 = 27.2%, and Q4 = 18.5%. A chi-squared test revealed a statistically significant deviation from the expected uniform distribution (χ^2^(3) = 9.81, *p* = 0.020), indicating the presence of an RAE. OR comparisons further showed an over-representation of athletes born in Q1 compared to Q4 (OR = 2.07) and a moderate over-representation in Q3 compared to Q4 (OR = 1.47). The comparison between Q2 and Q4 suggested a slightly reduced representation (OR = 0.87). These results indicate that athletes born earlier in the selection year were disproportionately represented in the Olympic karate cohort at Tokyo 2020 (Table 1).

### 3.2. Effect of Sex

For male athletes (*n* = 42), the birth quartile distribution was Q1 = 31.0%, Q2 = 19.0%, Q3 = 33.3%, Q4 = 16.7%. The chi-squared test did not show a statistically significant deviation from uniformity (χ^2^(3) = 3.52, *p* = 0.318). ORs indicated moderate over-representations in Q1 vs. Q4 (OR = 1.86) and Q3 vs. Q4 (OR = 2.00), but these differences were not significant (Table 1). For female athletes (*n* = 39), the distribution was Q1 = 46.2%, Q2 = 12.8%, Q3 = 20.5%, Q4 = 20.5%. A significant deviation from uniformity was observed (χ^2^(3) = 9.92, *p* = 0.019). ORs confirmed a strong over-representation in Q1 vs. Q4 (OR = 2.25) and an under-representation in Q2 vs. Q4 (OR = 0.63) (Table 1).

### 3.3. Effect of Discipline

For kata athletes (*n* = 21), the distribution was Q1 = 14.3%, Q2 = 23.8%, Q3 = 28.6%, and Q4 = 33.3%. No significant deviation was observed (χ^2^(3) = 1.67, *p* = 0.644), suggesting no evidence of RAE in kata. ORs also showed no over-representation in earlier quartiles: Q1 vs. Q4 (OR = 0.43), Q2 vs. Q4 (OR = 0.71), and Q3 vs. Q4 (OR = 0.86) (Table 1). When athletes were aggregated in semesters of birth, the distribution was not significantly different from uniform (χ^2^(1) = 1.19, *p* = 0.275), although 38.1% of athletes were born in S1 and 61.9% in S2 (OR = 0.62) (Table 2).

For kumite (*n* = 60), the distribution was markedly skewed: Q1 = 46.7%, Q2 = 13.3%, Q3 = 26.7%, Q4 = 13.3%. A chi-squared test revealed a highly significant deviation from uniformity (χ^2^(3) = 17.87, *p* < 0.001). ORs confirmed this effect: Q1 vs. Q4 (OR = 3.50), Q2 vs. Q4 (OR = 1.00), and Q3 vs. Q4 (OR = 2.00) (Table 1).

### 3.4. Combined Effect of Sex and Discipline

For male kumite athletes (*n* = 31), the distribution was Q1 = 35.5%, Q2 = 12.9%, Q3 = 35.5%, Q4 = 16.1%. The chi-squared test was not significant (χ^2^(3) = 5.52, *p* = 0.138), suggesting no strong evidence of RAE in this subgroup. However, ORs suggested moderate over-representations in Q1 vs. Q4 (OR = 2.20) and Q3 vs. Q4 (OR = 2.20) (Table 1). When athletes were aggregated in semesters of birth, the distribution was balanced (S1 = 48.4%, S2 = 51.6%), χ^2^(1) = 0.03, *p* = 0.857, OR = 0.94. For female kumite athletes, male and female kata athletes, the distributions in quartiles of birth can be observed in Table 1. When aggregated in semesters of birth, female Kumite athletes showed a significant RAE (S1 = 72.4%, S2 = 27.6%), χ^2^(1) = 5.83, *p* = 0.016, OR = 2.62.

For male Kata athletes, there was no difference between semesters (S1 = 54.5%, S2 = 45.5%), χ^2^(1) = 0.09, *p* = 0.763, OR = 1.20, while for females, analysis was not performed due to the low number of athletes per semester (Table 2).

### 3.5. Probability of Winning a Medal and Birthdate

A binary logistic regression using S2 as the reference category showed no statistically significant effect on medal attainment (OR = 0.49, 95% CI [0.20, 1.21], *p* = 0.125). This indicates that, at the semester level, no RAE was observed in the overall Olympic karate cohort. Table 3 presents the numbers and frequencies of medalists and non-medalists for the overall sample and subcategories.

## 4. Discussion

The present study investigated the presence of the RAE in Olympic karate for the first time, analyzing the distribution of birth quartiles among athletes competing at the Tokyo 2020 Games. The main findings revealed a significant overall RAE, particularly pronounced in kumite and among female athletes. No significant effect was detected in kata, where a non-significant inverse tendency emerged, nor among male athletes. These results confirm our initial hypothesis that athletes born earlier in the selection year would show a selection advantage. Moreover, this effect was more pronounced in kumite, the discipline with higher physical and perceptual–motor demands, which may be more sensitive to selection mechanisms associated with the RAE.

Previous research has reported mixed evidence of the RAE across combat sports. In Olympic taekwondo, no systematic effect has been observed, a finding that has been partly attributed to the historically limited global competitive depth of the sport, despite its strong cultural and institutional prominence in Korea [16]. By contrast, karate is practiced worldwide, with a broad base of participants and long-standing international structures. Although Tokyo 2020 marked the first inclusion of the discipline in the Olympic program, the global popularity of the sport may have fostered a highly competitive selection environment, thereby favoring the emergence of RAE [16]. Nevertheless, assessing the popularity of a sport across countries and quantifying the number of registered practitioners remains challenging, and such considerations should be interpreted with caution.

Sex differences in the manifestation of RAE have traditionally shown stronger effects among male athletes, with earlier studies often reporting little or no evidence in females [9]. This pattern has commonly been explained by the higher levels of participation and competition in men’s sport, where selection pressure accentuates the advantages of relative age [30,31]. However, our findings diverge from this trend, revealing a pronounced RAE among female karate athletes but only a non-significant tendency among males. This result aligns with more recent research suggesting that RAE is becoming increasingly visible in female sports, including combat sports [17,32,33]. The growing popularity of female competition, together with increased investment, media visibility, and improved training environments, likely amplifies selection pressures, thereby favoring relatively older athletes. it should be considered that gender norms have evolved markedly over the last decades, progressively expanding sporting opportunities for females (e.g., the inclusion of female boxing competitions at the XXX Olympic Games). Even in countries with more restrictive laws and traditions, institutional policies have increasingly promoted gender equity in sport [34]. As female combat sports gain visibility through increased federative support and media exposure, a growing number of young females are encouraged to participate and to engage in early, sport-specific training. However, access to high-level competitive pathways may still depend on social and institutional support structures, which can favor relatively older athletes perceived as more mature or ready for competition. Moreover, in combat sports such as karate, physical robustness and perceptual–motor skills may confer an even clearer advantage in selection process for females, where relative differences in strength and body size can decisively affect the ability to neutralize an opponent in direct confrontation [35]. It has been suggested that natural developmental phases, which differ between males and females, may contribute to intrinsic differences in speed and agility profiles between sexes [21]. Considering that female maturation occurs earlier, this may reinforce the selection advantage associated with the RAE during the growth spurt phase, causing it to emerge earlier than in males and to persist into the elite level.

Disciplinary differences further highlight the specificity of RAE in karate. Given the contrasting technical and physical demands of kata and kumite, the mechanisms through which RAE may operate are expected to differ between the two disciplines. While kumite exhibited a strong and significant RAE, no effect was found in kata, where the distribution even suggested an inverse trend. This contrast is likely rooted in the distinct performance demands of the two disciplines. Kumite involves direct physical confrontation, where strength, speed, and body size can provide immediate advantages, and relatively older athletes may be preferentially selected. However, kumite also places greater demands on speed, reactivity, timing, and perceptual–motor coordination. These attributes, often linked to neuromuscular and cognitive maturation rather than pure muscular strength, may confer a decisive selection advantage in rapid attack–counterattack exchanges. Thus, in karate, relative age advantages may emerge not only from physical robustness but also because earlier development of coordination, reaction ability, and tactical anticipation can influence selection and training opportunities. Indeed, relative age selection advantage has been linked to change of direction, agility, and reaction time, but also motor learning and creativity in movement and sport [36,37,38,39]. By contrast, kata is judged on technical precision, balance, and aesthetic expression rather than direct dominance over an opponent. In this respect, kata shares similarities with artistic or aesthetic sports such as gymnastics and dance, where inverse RAEs have sometimes been reported [40]. It is also plausible that relatively younger athletes, who may find themselves at a disadvantage in kumite due to their lower physical maturity, tend to specialize in kata, where technical and aesthetic skills can compensate for physical differences. Such a self-selection process could amplify the RAE in kumite while contributing to its absence, or even reversal, in kata. In these contexts, a lighter body structure, greater flexibility, and a more compact physique, traits more commonly associated with relatively younger athletes, may be valued. Thus, even though this finding should be interpreted cautiously, the absence of RAE in kata may reflect the lesser importance of physical superiority compared to technical and artistic mastery.

The absence of a significant RAE among male athletes in this study is more difficult to interpret. One possibility is that, despite the global popularity of karate, the pool of elite male competitors at the Olympic level is relatively narrow, with strong filtering already occurring at earlier developmental stages. In such a scenario, only the most talented, physically prepared, and psychologically equipped athletes, regardless of relative age, may reach the top level, thereby attenuating the observable effect at the Olympic stage. Another explanation may lie in the balance between technical and tactical skills, which in high-level male kumite could partially compensate for physical advantages linked to relative age. This interpretation is consistent with research suggesting that RAE is most pronounced in youth and developmental categories and tends to diminish in adulthood as performance becomes increasingly shaped by experience and skill rather than chronological maturity [3,9]. In line with this view, a previous study using a Brazilian database of youth combat athletes found that RAE occurred in karate categories U-14 and U-16, being stronger in males but similar between sexes in the kumite discipline [21]. Furthermore, another study conducted at the Spanish national championships reported a clear RAE in youth and junior categories, but no effect among senior athletes of either sex [23]. These findings support the idea that because male maturation occurs later, RAE related to growth spurts may also emerge later, allowing relatively younger male athletes more time to emerge. In contrast, the finding of a comparable RAE between sexes in kumite at the youth level in Brazil underlines how, in this discipline, physical profile is more relevant [21].

When considered within the broader landscape of combat sports, these findings reinforce the notion that RAE is not uniform across disciplines but strongly influenced by the structure of competition and the underlying performance demands. In sports such as wrestling and judo, where physical confrontation and weight categories play a central role, consistent RAEs have been observed, particularly among men [14,15]. In taekwondo, however, the evidence remains inconclusive, likely reflecting lower competitive density at the international level [16]. On the other hand, Kim et al. suggested that an RAE was present in boxing, especially in young females [17]. Taken together, these findings suggest that RAE tends to be more pronounced in grappling-based combat sports and less evident in striking disciplines, at least among male athletes. It is plausible that in grappling sports, where body mass and strength play a decisive role, physical maturity exerts a stronger influence on performance, particularly in men. However, this hypothesis has yet to be directly tested. Our results suggest that karate aligns more closely with striking combat sports and, especially among females, RAE can shape selection processes and thereby influencing which athletes are more likely to progress to the elite level. At the same time, the absence of an effect in kata highlights how disciplines within the same sport may diverge depending on whether performance relies on direct opposition or on technical and aesthetic criteria. This supports the idea that RAEs in combat sports should be understood not as a homogeneous phenomenon, but rather as a context-dependent outcome shaped by the interaction between participation levels, selection pressure, and the specific demands of each discipline.

In contrast to the clear evidence of RAE in participation, the logistic regression analyses did not reveal any significant advantage for relatively older athletes on the likelihood of winning a medal, either in the overall sample or when restricting the analysis to kumite athletes. Furthermore, in the overall sample, lower odds of medaling for athletes born in S1 were observed compared to those born in S2. This outcome is mainly due to kata specialists: indeed, while the proportion of medalists is almost balanced in kumite, only one male medalist and no female medalists were observed in S1 for kata. These results suggest that while relative age may influence the selection pathway up to Olympic qualification, once athletes reach the elite level of Olympic competition, performance outcomes are no longer associated with those factors that shaped the earlier selection process. These finding echoes previous evidence in other sports, where RAE tends to be more pronounced in the processes of talent identification and selection, but less predictive of success in elite performance contexts [9]. Indeed, although differences in maturation rate may influence body size, composition, and skills, thus favoring early maturers during youth, once maturation is complete, such advantages appear to dissipate [41,42,43,44,45].

From a practical perspective, these findings underscore the importance of considering relative age in talent development systems for combat sports. Coaches and federations should be aware that selection mechanisms may inadvertently favor relatively older athletes, particularly in kumite and among females, potentially excluding younger athletes who could succeed given time and appropriate support. Misinterpreting RAE may not only result in biased talent identification and selection errors but also have long-term implications for athletes’ health and well-being. Recent perspectives have emphasized that combat sports environments can entail both opportunities and risks, and that safeguarding athlete health requires careful attention to how competitive structures are organized [13,46]. For instance, it has been shown that less mature athletes often display poorer health-related indices compared to their more mature peers of the same chronological age, particularly in terms of adiposity and cardiometabolic risk [47,48]. Supporting these athletes adequately throughout their sporting pathway is therefore crucial to reduce dropout and safeguard their well-being. Measures such as bio-banding, flexible age-grouping, or longitudinal monitoring of athlete progression may help mitigate these biases and foster more equitable opportunities [8].

This study has some limitations. The sample was restricted to a single Olympic edition, which, although globally representative, captures only a snapshot of elite karate and does not account for longitudinal dynamics. Moreover, the relatively small number of kata competitors reduced the statistical power to detect effects. While the total sample size was sufficient for robust chi-squared and odds ratio analyses, the relatively small number of kata athletes, and especially some sex-based subgroups, limited the statistical power and precluded further analyses. Moreover, for certain kumite subgroups, the number of athletes per quartile fell below the recommended threshold of five cases per cell, which may reduce the reliability of the statistical estimates and should be taken into consideration when interpreting these findings [49]. Aggregating the quartile of birth into semesters allowed for a more robust analysis, although it was less detailed. For all these reasons, the findings of the present study may not generalize to lower levels of karate competition and need to be interpreted with caution. Nevertheless, the study also presents notable strengths. To the best of our knowledge, this is the first investigation of RAE in Olympic karate, providing novel insights into a sport that was introduced to the Olympic program for the first time at the Tokyo 2020 Games. Furthermore, the dataset represents an elite cohort of athletes who had reached the highest level of international competition, ensuring that the findings reflect the dynamics of selection and participation at the very top of the performance pyramid. Future research should broaden the scope by including multiple levels of competition (youth, junior, senior) across both national and international contexts, while also examining longitudinal trajectories of athletes. Given that RAE tends to exert its strongest influence during talent identification and selection processes yet appears less predictive of success at the elite level, such comprehensive approaches are needed to clarify how RAE shapes entry pathways into elite karate. Moreover, longitudinal analyses would allow researchers to examine whether and how the early advantages associated with relative age persist, diminish, or reverse over time, thereby offering deeper insights into the complex interactions between RAE, performance outcomes, and long-term career development.

## 5. Conclusions

This study provides the first evidence of the relative age effect in Olympic karate, using data from the Tokyo 2020 Games. The analyses revealed a significant overall RAE, with stronger effects in kumite and among female athletes, while no significant effect was observed in kata or among males. These patterns suggest that selection and developmental mechanisms in karate may differ according to sex and event type. Logistic regression analyses indicated that relative age did not predict medal success, suggesting that performance at the Olympic level is primarily determined by technical, tactical and psychological competencies rather than birth quartile. Collectively, these findings underscore the importance of considering RAE in the earlier stages of athlete identification and development, where selection biases may shape access to high-level opportunities. Enhancing awareness of such biases among coaches, talent scouts, and sport federations could contribute to more equitable selection processes and foster long-term athlete development. Future research should adopt longitudinal and multilevel approaches to clarify how relative age interacts with training exposure, competitive experience, and performance trajectories across an athlete’s career.

## Figures and Tables

**Table 1:** Birth quartile distributions, chi-squared tests, and odds ratios for the total sample and subgroups (sex and discipline).

Discipline	Group	Q1 (n/%)	Q2 (n/%)	Q3 (n/%)	Q4 (n/%)	χ^2^	*p*-Value	OR Q1/Q4	OR Q2/Q4	OR Q3/Q4
Overall										
	Overall	31 (38%)	13 (16%)	22 (27%)	15 (19%)	9.81	0.020	2.07	0.87	1.47
	M	13 (31%)	8 (19%)	14 (33%)	7 (17%)	3.52	0.318	1.86	1.14	2.00
	F	18 (46%)	5 (13%)	8 (21%)	8 (21%)	9.92	0.019	2.25	0.63	1.00
Kata										
	Overall	3 (14%)	5 (24%)	6 (29%)	7 (33%)	1.67	0.644	0.43	0.71	0.86
	M	2 (18%)	4 (36%)	3 (27%)	2 (18%)	--	--	--	--	--
	F	1 (10%)	1 (10%)	3 (30%)	5 (50%)	--	--	--	--	--
Kumite										
	Overall	28 (47%)	8 (13%)	16 (27%)	8 (13%)	17.87	<0.001	3.50	1.00	2.00
	M	11 (36%)	4 (13%)	11 (36%)	5 (16%)	5.52	0.138	2.20	0.80	2.20
	F	17 (59%)	4 (14%)	5 (17%)	3 (10%)	--	--	--	--	--

The table reports the number of participants (n), the percentage frequency (%), and the values of the Chi-square test (χ^2^), statistical significance (*p*-value), and Odds Ratios (OR) for the different birth quartiles (Q1: January–March; Q2: April–June; Q3: July–September; Q4: October–December) and subgroups (Overall: entire sample; M: males; F: females). Chi-square χ^2^ results for Kata and Kumite male athletes are approximate (one expected cell frequency < 5).

**Table 2:** Birth semester distributions, chi-squared tests, and odds ratios for subgroups (sex and discipline).

Discipline	Group	S1 (n/%)	S2 (n/%)	χ^2^	*p*-Value	OR (S1/S2)
Kumite						
	M	15 (48.4%)	16 (51.6%)	0.03	0.857	0.94
	F	21 (72.4%)	8 (27.6%)	5.83	0.016	2.62
Kata		8 (38.1%)	13 (61.9%)	1.19	0.275	0.62
	M	6 (54.5%)	5 (45.5%)	0.09	0.763	1.20
	F	2 (20.0%)	8 (80.0%)	--	--	--

The table reports the number of participants (n), the percentage frequency (%), and the values of the Chi-square test (χ^2^), statistical significance (*p*-value), and Odds Ratios (OR) for the different birth semesters (S1: January–June; S2: July–December) and subgroups: M (males) and F (females).

**Table 3:** Birth semester distributions of medalists and non-medalists for the overall sample, sex, and discipline.

Discipline	Group	Semester of Birth	Medalists (n/%)	Non-Medalists (n/%)
Overall				
	Overall	S1	14 (31.8%)	30 (68.2%)
		S2	18 (48.6%)	19 (51.4%)
	M	S1	7 (33.3%)	14 (66.7%)
		S2	9 (42.9%)	12 (57.1%)
	F	S1	7 (30.4%)	16 (69.6%)
		S2	9 (56.2%)	7 (43.8%)
Kata				
	Overall	S1	1 (12.5%)	7 (87.5%)
		S2	7 (53.8%)	6 (46.2%)
	M	S1	1 (16.7%)	5 (83.3%)
		S2	3 (60.0%)	2 (40.0%)
	F	S1	0 (0.0%)	2 (100.0%)
		S2	4 (50.0%)	4 (50.0%)
Kumite				
	Overall	S1	13 (36.1%)	23 (63.9%)
		S2	11 (45.8%)	13 (54.2%)
	M	S1	6 (40.0%)	9 (60.0%)
		S2	6 (37.5%)	10 (62.5%)
	F	S1	7 (33.3%)	14 (66.7%)
		S2	5 (62.5%)	3 (37.5%)

The table reports the number of participants (*n*) and the percentage frequency (%) of medalists and non-medalists for the different birth semesters (S1: January–June; S2: July–December) and subgroups: M (males) and F (females).

## Data Availability

The data used in this study are publicly available from open-access sources, including the official Tokyo 2020 Olympic Games website and the World Karate Federation athlete profiles [24,25]. No new data were created or collected by the authors.

## Supplementary Materials

The following supporting information can be downloaded at: https://www.mdpi.com/article/10.3390/jfmk10040456/s1. Table S1. STROBE Statement—Checklist of items that should be included in reports of cross-sectional studies.

## Abbreviations

The following abbreviations are used in this manuscript:

RAE	Relative Age Effect
ORs	Odds Ratios
CIs	Confidence Intervals
Q1–Q4	Birth quartile; Q1: January–March; Q2: April–June; Q3: July–September; Q4: October–December
S1–S2	Birth Semesters; S1: January–June; S2: July–December
